# IL-13 Derived Type 2 Innate Lymphocytes Ameliorates Cardiomyocyte Apoptosis Through STAT3 Signaling Pathway

**DOI:** 10.3389/fcell.2021.742662

**Published:** 2021-09-20

**Authors:** Ting Hong, Saiqi Li, Xiaoyu Guo, Yazhong Wei, Jingjing Zhang, Xiaohui Su, Miao Zhou, Haizhen Jin, Qing Miao, Lei Shen, Minfang Zhu, Bin He

**Affiliations:** ^1^Department of Critical Care Medicine, Shanghai Chest Hospital, Shanghai Jiao Tong University, Shanghai, China; ^2^Shanghai Institute of Immunology, Shanghai Jiao Tong University School of Medicine, Shanghai, China; ^3^Department of Anesthesiology and Surgical Intensive Care Unit, Xinhua Hospital, Shanghai Jiaotong University School of Medicine, Shanghai, China; ^4^Central Laboratory, Shanghai Chest Hospital, Shanghai Jiao Tong University, Shanghai, China; ^5^Departments of Anesthesiology, Shanghai Chest Hospital, Shanghai Jiao Tong University, Shanghai, China

**Keywords:** sepsis, cardiomyocyte apoptosis, IL-13, ILC2, stat3

## Abstract

The involvement of cardiomyopathy during sepsis means higher mortality and prolonged length of hospital stay. Many efforts have been made to alleviate the apoptosis of cardiomyocytes in sepsis. The huge potential of IL-13 in tissue repair has attracted increasing attention. In the present study, we used LPS-treated mice or primary cardiomyocytes as a sepsis model to explore the anti-apoptotic ability of IL-13. It was found that an increased level of exogenous IL-13 was beneficial to the recovery of heart function in sepsis, and this anti-apoptotic effect of IL-13 was probably through enhancing the phosphorylation of STAT3 Ser727. In addition, we identified that the heart protective effect of IL-13 was associated with type 2 innate lymphocytes (ILC2). All these findings may provide a potential promising treatment for sepsis-induced cardiomyopathy.

## Introduction

Although sepsis-induced cardiac injury has been acknowledged as a leading cause of death in the intensive care unit (ICU), there is still limited knowledge about the underlying mechanism (Joseph et al., 2017; Ehrman et al., 2018). It is generally recognized that immoderate and excessive host immune responses play a central role in reducing the reactivity and exacerbating the apoptosis of cardiomyocytes (Kamisoglu et al., 2015; Akama et al., 2021). It is necessary to understand the variability of host immune responses, knowing that it may help decrease apoptosis of cardiomyocytes and develop new strategies for the treatment of sepsis-induced cardiac injury (Zechendorf et al., 2020).

IL-13 is a representative cytokine of type 2 immunity and participates in host protection through promoting tissue repair and controlling inflammation progress (Neill et al., 2010; Gause et al., 2013; Gieseck et al., 2018). IL-13 is a crucial regulator of cardiomyocyte fate by reversing transcription during heart generation, demonstrating the cardiac protection of IL-13 (O’Meara et al., 2015). Although IL-13 shows great therapeutic potential in the treatment of cardiovascular diseases (Hofmann et al., 2014; Wodsedalek et al., 2019; Zlatanova et al., 2019), the role of IL-13 in protecting the septic cardiomyopathy remains to be further understood.

In this study, we investigated the expression level of IL-13 at different times after intraperitoneal (i.p.) injection of lipopolysaccharide (LPS), knowing that it could simulate endotoxemia during the sepsis state (Ehrman et al., 2018). Subsequently, we explored the protection mechanism of IL-13 in sepsis-induced cardiomyopathy through inhibiting the apoptosis of cardiomyocytes, and found that type 2 innate lymphocyte (ILC2) was the main source of IL-13. These results demonstrated that IL-13 played a significant cardioprotective role in sepsis-induced cardiac injury, which may provide a new remedy for clinical translation.

## Materials and Methods

### Materials and Regents

Materials and reagents used in this study were lipopolysaccharide (LPS), DNase I and collagenase (Sigma, United States); 0.25% trypsin, 0.25% trypsin-EDTA and fetal bovine serum (FBS) (Gibco, United States); Dulbecco’s Modified Eagle’s Medium (DMEM) and D-Hank’s balanced salt solution (D-Hank’s) (Cytiva, United States); fluorescence-labeled antibodies (Biolegend, United States; details are listed in Supplementary Table 1); ermeabilization buffer, MitoTracker^TM^ Deep Red and DAPI (Invitrogen, United States); antibody of cleaved caspase 3, STAT3, phospho-STAT3 (Tyr705), phospho-STAT3 (Ser727), and β-actin (CST, United States); IL-13 and IL-13Rα1 antibodies (Santa Cruz Biotechnology, United States); primers (BioTNT, China); PVDF membrane (Millipore, United States); reactive oxygen species assay kit (DCFH-DA), red blood cell lysis buffer, cell mitochondria isolation kit and antibody of α-tubulin (Beyotime, China); GentleMACS C tubes (Miltenyi Biotech, United States); *Invivo* anti-CD90.2 (Bioxcell, Italy); FITC-Annexin V (BD, United States); RNA purification kit (EZBioscience, United States).

### Cell Culture and Treatment

Primary cardiomyocytes were extracted from the rat hearts as previously described (Hong et al., 2020). In brief, the hearts were isolated from 2-day-old SD rats, washed with D-Hank’s, cut into small pieces and incubated with 0.1% trypsin for 15 min to acquire single cardiomyocyte suspension. Then, low-glucose DMEM containing 10% FBS was added to the cardiomyocytes to terminate the digestion. This step is repeated five times until no lumps are visible. Finally, the primary cardiomyocytes were seeded into culture dishes at a density of 3–5 × 10^5^ cells mL-1 in low-glucose DMEM containing 10% FBS for further use.

### Experiment With the Sepsis-Induced Cardiac Injury Model

Sepsis-induced cardiac injury was established in animal and cell models.

Male C57BL/6 mice weighing 25–28 g (GemPharmatech Co., Jiangsu, China) were raised in a specific pathogen-free (SPF) environment in Shanghai Chest Hospital (Shanghai, China). All experimental procedures were approved and supervised by the Ethics Committee of the said hospital. 20 mg/kg LPS was injected i.p. to C57BL/6 LPS mice to establish LPS-induced cardiac injury.

In the cell model, primary cardiomyocytes were incubated with low-glucose DMEM containing 10% FBS and 10 μg/ml LPS to mimic endotoxin-induced cell damage.

### RNA Extraction

RNA was extracted using the RNA purification kit. 40 mg heart tissue was put into a 1.5 ml centrifuge tube and added with 300 μl lysis buffer. Then, the tissue was homogenized in a rotor-stator homogenizer and centrifuged at 12,000 g for 2 min and mixed with an equal volume of ethanol. The mixture was added to the RNA column to bind RNA to the membrane, centrifuged at 4,000 g for 1 min, and after addition of 500 μl wash buffer into the spin column, centrifuged again at 12,000 g for 1 min. The spin column was transferred to a new RNase free tube, to which 20 μl elution buffer was added into the center of spin column at room temperature. Finally, the sample was centrifuged at 12,000 g for 1 min to remove the spin column.

### Real-Time RT-PCR

Total RNA extracted from the mice heart was converted to cDNA for quantitative PCR (qPCR) using 4 × Reverse Transcription Master Mix Kits (EZBioscience, United States). The expression level of GAPDH was quantitated as an internal control according to the 2 × SYBR Green qPCR Master Mix (EZBioscience, United States) manufacturer’s instructions. The primers used in the work were purchased from BioTNT, Shanghai.

### Preparation of the Single-Cell Suspension and Flow Cytometry Analysis

The C57BL/6 mouse hearts were made into single-cell suspension via gentleMACS^TM^ Octo Dissociator for flow cytometry analysis. Briefly, the fresh mouse heart was washed with cold PBS to remove peripheral blood cells and cut into small pieces. The heart tissue was digested in D-Hank’s containing 1 mg/ml collagenase II and 10 μg/ml DNase I under the program of 37_mmu Adult Heart Procedure. Then, red blood cells (RBCs) were lysed and washed with cold PBS twice.

After 200 mesh screen filtration, the cells were ready for staining. Firstly, cells were incubated with Live/Dead dye for 15 min at room temperature, surface stained using the standard protocol for 15 min at room temperature, fixed, permeabilized with permeabilization buffer at 4°C overnight, stained with corresponding antibodies for another 30 min, washed with PBS, resuspended in 100 μl PBS, and finally detected by BDFACSFortessa 4-Laser (BD, United States).

### Protein Extraction and Western Blotting Assay

Protein extraction and Western blotting were performed as previously described (Virga et al., 2021). Briefly, the protein sample (30 μg) was electrophoresed in SDS-PAGE and transferred to the PVDF membrane. The blots were incubated with the primary antibody at 4°C overnight, followed by incubation with the HRP-conjugated secondary antibody. The Western blot bands were detected by A6100 ECL imaging system (GE, United States) and quantitated with Image J software by measuring the intensity of each band compared with the internal control band.

### Cell Apoptosis Assay

Apoptosis and morphology were detected simultaneously by fluorescence microscopy. Briefly, primary cardiomyocytes were incubated with FITC-labeled Annexin V diluted by the banding buffer at 37°C for 15 min. Then cells were washed with PBS and added with Mitotracker at 37°C for another 30 min. The stained cells were washed with PBS for 3 times and detected.

### Deletion of Innate Lymphoid Cells

Innate lymphoid cells (ILCs) of C57BL/6 mice were deleted by i.p. injection of CD90.2 antibody at a dose of 12 mg/kg for 4 times at a 4-day interval. The deletion efficiency of ILC2 was determined by flow cytometry.

### Data Analysis and Statistics

All experiments were repeated with three or more biological replicates. Data were analyzed by PRISM software (Graphpad, United States) and presented as the mean ± standard deviation (SD). One-way ANOVA was used to analyze differences between multiple groups and the two-tailed Student’s *t*-test was used for comparison between two groups. *P*-value < 0.05 was considered statistically significant.

## Results

### IL-13 Underwent Changes in a Time-Dependent Manner After Intraperitoneal Injection of Lipopolysaccharide in Mice

**FIGURE 1:**
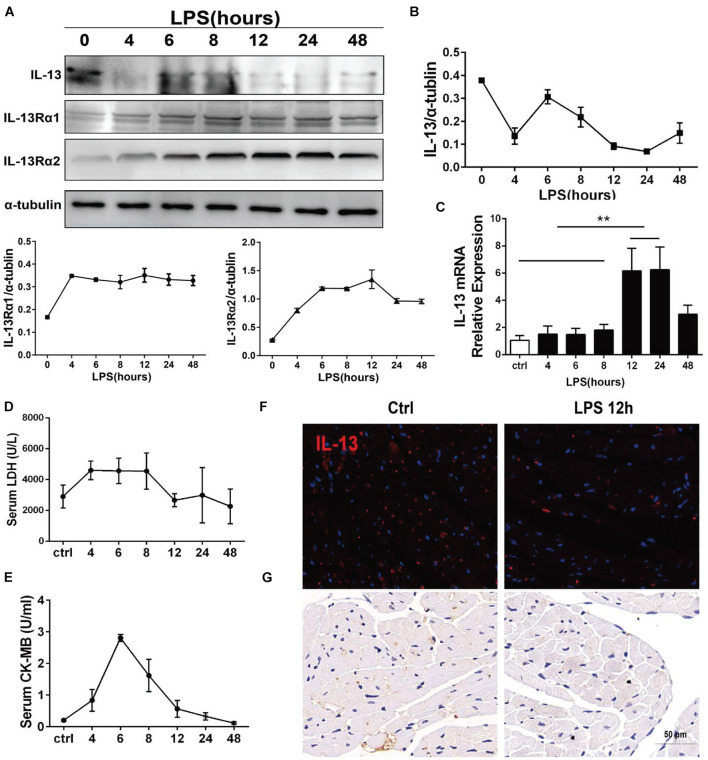
IL-13 underwent changes in a time-dependent change after i.p. injection of LPS in mice. **(A)** Determination of IL-13, IL-13Rα1, and IL-13Rα2 relative expressions in the myocardial tissue by Western blot after i.p. injection of LPS in mice. **(B)** Quantification of **(A)**. **(C)** Detection of mRNA expression of IL-13 in the myocardial tissue by RT-qPCR. **(D)** Serum LDH change. **(E)** Serum CK-MB change. **(F,G)** Immunofluorescence and Immunohistochemical images of IL-13 in the mouse myocardium after 12-h exposure to LPS, Scale bar = 50 μm. All the experiments were repeated three times. Data were expressed as the mean ± SD (*n* = 3). **p < 0.01.

The protein expression of IL-13 in the mouse myocardium was continuously monitored by Western blot within 48 h after i.p. injection of LPS, showing a trend of rapid decline, transient rise and continuous low expression. At the same time, the two type receptors of IL-13, IL-13Rα1, and IL-13Rα2 increase steadily (Figures 1A,B). The mRNA of IL-13, and IL-13Rα1 showed similar changes in a time-dependent manner by qPCR (Figure 1C and Supplementary Figure 1). Although the transcription level of IL-13 increased continuously, the protein expression of IL-13 did not remain at a high level, indicating that IL-13 may be consumed heavily. Lactate dehydrogenase (LDH) and creatine kinase isoenzymes (CK-MB) as markers of myocardial injury were detected in the mouse plasma within 48 h after i.p. injection of LPS. It was found that the time-dependent change of LDH and CK-MB was related to the expression of IL-13 (Figures 1D,E). The critical turning point was observed at 12 h after LPS exposure, when the expression of IL-13 protein in the mouse myocardial tissue was reconfirmed to be significantly decreased as shown by immunofluorescence and histochemistry staining (Figures 1F,G). In addition, the level of IL-13 in the mouse plasma was also decreased significantly at this time point, suggesting that IL-13 may play an important role in septic cardiomyopathy.

### IL-13 Alleviated Lipopolysaccharide-Induced Cardiac Dysfunction and Reduced Apoptosis in Mice

**FIGURE 2:**
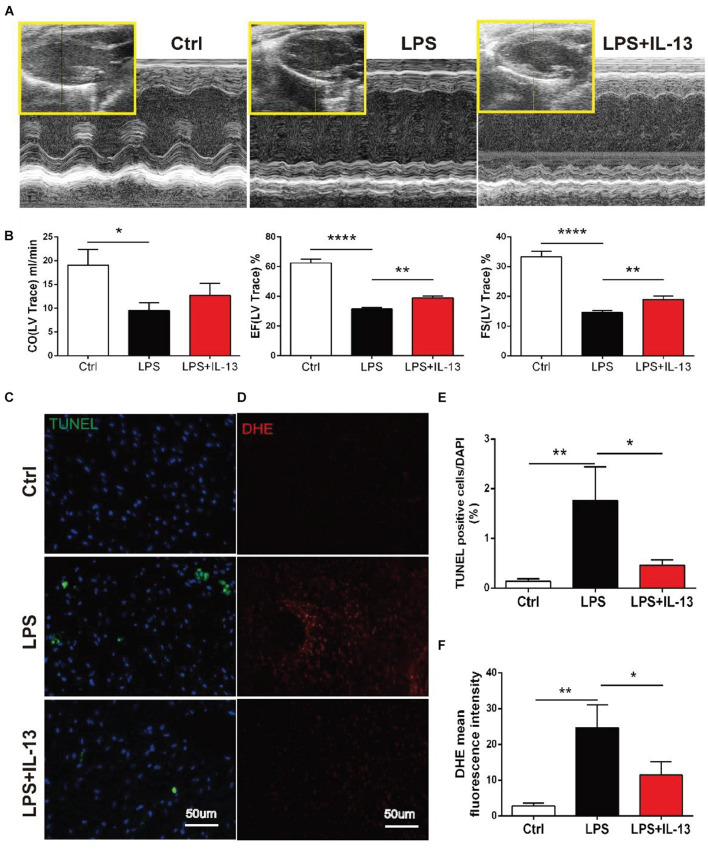
IL-13 alleviated LPS-induced cardiac dysfunction and reduced apoptosis in mice. **(A)** Representative images of M-mode echocardiography. **(B)** Quantification of cardiac output (CO), ejection fraction (EF) and fractional shortening (FS) by echocardiography. **(C)** Representative TUNEL staining in the cardiac tissue, Scale bar = 50 μm. **(D)** Representative images of dihydroethidium (DHE) in the cardiac tissue, Scale bar = 50 μm. **(E)** Percentage of TUNEL-positive nuclei. **(F)** DHE oxidation values represent the mean fluorescent intensity (MFI). Data were expressed as the mean ± SD (*n* = 6). *p < 0.05, **p < 0.01, ****p < 0.0001.

To explore the role of IL-13 in LPS-induced myocardial injury, mice injected i.p. with LPS were treated with recombinant IL-13 (rIL-13, 100 μg/kg). As shown in Figures 2A,B, rIL-13 effectively recovered the myocardial contractile function induced by LPS. Specifically, Cardiac output (CO), Ejection fraction (EF), and Fractional shortening (FS) all recovered in varying degrees. These results suggest that IL-13 could improve cardiac function. The production of reactive oxygen species (ROS), which is known to be closely related to myocardial injury, is shown in the images of dihydroethidium (DHE). As shown in Figures 2D,F, IL-13 significantly reduced the level of DHE oxidation induced by LPS. Furthermore, the degree of myocardial apoptosis was detected by TUNEL, and the result showed that IL-13 effectively reduced myocardial apoptosis (Figures 2C,E).

### IL-13 Alleviated Lipopolysaccharide-Induced Cardiomyocyte Apoptosis

**FIGURE 3:**
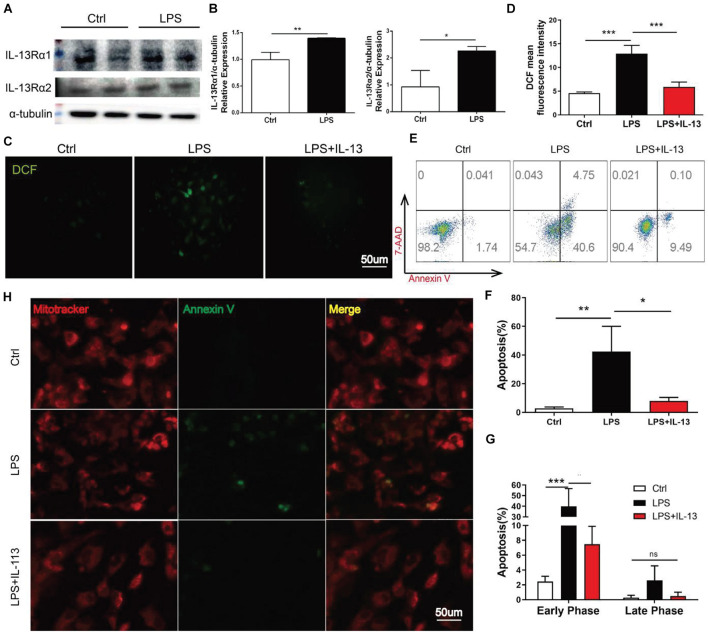
IL-13 alleviated LPS-induced cardiomyocyte apoptosis. **(A)** Determination of IL-13Rα1 and IL-13Rα2 relative expressions in the primary cardiomyocytes by Western blot. **(B)** Quantification of **(A)**. **(C)** DCF fluorescent images, Scale bar = 50 μm. **(D)** DCF mean fluorescent intensity. **(E)** Flow cytometry analysis of apoptosis. **(F,G)** Quantification of apoptosis. **(H)** Cardiomyocytes stained with mitoitrcacker (red) and Annexin V(green), Scale bar = 50 μm. Data were expressed as the mean ± SD (*n* = 3). *p < 0.05, **p < 0.01, ***p < 0.001.

To identify the effect of IL-13 on cardiomyocytes, the primary cardiomyocytes were extracted as the experimental object *in vitro*. Firstly, the two receptors of IL-13 were detected by Western blot in the primary myocardium. The results showed that both receptors existed in the primary myocardium and increased upon LPS stimulation, with the increase of IL-13Rα1 more significant (Figures 3A,B). The production of reactive oxygen promoted DCFH transformation into DCF, and the mean fluorescence intensity of DCF was measured to represent the level of ROS in primary myocardiocytes. As shown in Figures 3C,D, cardiomyocytes ROS production induced by LPS was effectively inhibited by IL-13. Next, apoptosis was detected by flow cytometry. Compared with LPS treatment alone, the apoptosis of primary cardiomyocytes exposed to IL-13 (50 ng/ml) was significantly decreased, involving both early and late stages (Figures 3E–G). In the apoptosis process, phosphatidylserine (PS) was translocated from the inner to the outer leaflet of the plasma membrane, thus exposing PS to the external cellular environment. Annexin V labeled with a fluorophore was used to identify apoptotic cells by binding to PS exposed on the outer leaflet. As presented in Figure 3H, LPS accelerated PS exposure to the external environment of cells and weakened mitochondrial fluorescence. On the contrary, IL-13 effectively reversed this process.

### The Transcription Level of STAT3 but Not STAT6 Was Altered in the Signaling Pathway Affected by IL-13

**FIGURE 4:**
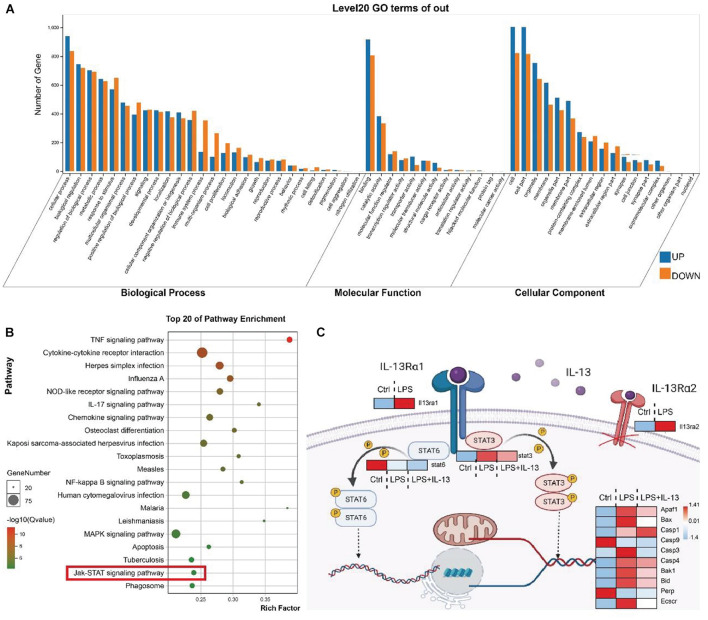
The transcription level of STAT3 but not STAT6 was altered in the signaling pathway affected by IL-13. **(A)** GO analysis covered three domains including the biological process, molecular function and cellular component. The transcription of the heart tissue in mice receiving i.p. injection of LPS vs. control mice. **(B)** KEGG analysis revealed the most important signaling pathways involved in gene involvement. The transcription of the heart tissue of mice receiving i.p. injection of LPS vs. control mice. **(C)** Illustration of the downstream key genes in the heart tissue regulated by IL-13 as shown by RNA sequencing (RNA-seq). Data are presented as heatmap (log_2_-fold change). Red indicates higher expression, and blue indicates lower expression (*n* = 6).

Heart tissues of LPS treated or untreated mice were collected for RNA sequence (RNA-seq). Gene Ontology (GO) analysis showed that a number of genes underwent changes in the three fields including the biological process, molecular function and cell composition with sepsis induced via i.p. injection of LPS (Figure 4A). The signaling pathways affected by LPS were further analyzed with Kyoto Encyclopedia of Genes and Genomes (KEGG) (Figure 4B). To determine the mechanism by which IL-13 reduced myocardial apoptosis caused by LPS, rIL-13 treatment of LPS-induced myocardial tissue in septic mice was included in RNA-seq. Stat6 is a typical transcription factor that mediates downstream transcription activated by IL-13 in immune cells. In other cell types, it is described that Stat3 is activated by Jak signaling downstream of IL-13Rα1. In the mouse myocardium, the Jak-STAT signaling pathway was identified by RNA-seq as being significantly changed after LPS stimulation (Figure 4B). As shown in Figure 4C, the transcription of IL-13Rα1 and Stat3 was highly expressed under the action of LPS, while the transcription of Stat6 changed slightly, suggesting that IL-13 reduced apoptosis in the myocardial tissue through the transcription factor Stat3. IL-13Rα2 is a decoy receptor, and we did not explore its effect in this study.

### Effect of IL-13 on the Phosphorylation of STAT3 in Cardiomyocytes

**FIGURE 5:**
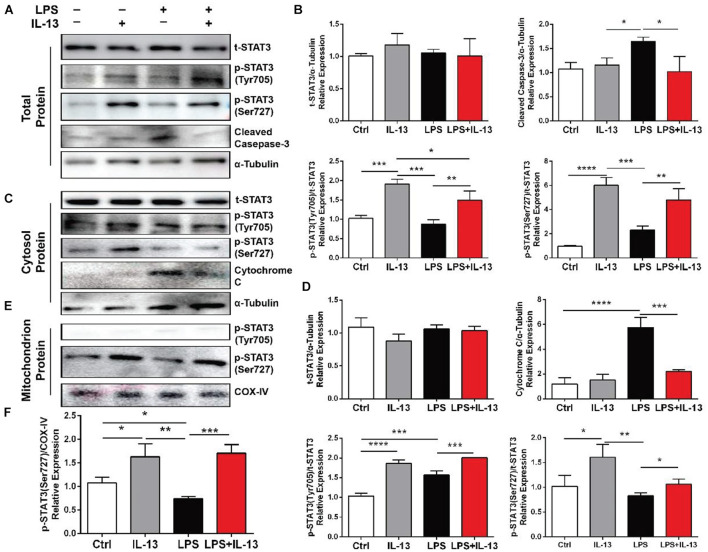
Effect of IL-13 on the phosphorylation of STAT3 in cardiomyocytes. **(A)** Western blot images of total protein expression. **(B)** Quantification of **(A)**. **(C)** Western blot images of the cytosol protein expression. **(D)** Quantification of **(C)**. **(E)** Western blot images of the mitochondrial protein expression. **(F)** Quantification of **(C)**. Data are expressed as the mean ± SD (*n* = 3). *p < 0.05, **p < 0.01, ***p < 0.001, ****p < 0.0001.

Total protein, cytosol protein and mitochondrial protein were extracted from the primary cardiomyocytes and analyzed by Western blot. It was found that the LPS-induced increase of cleaved Caspase-3 was effectively inhibited by IL-13 (Figures 5A,B). Meanwhile, the expression of STAT3 Serine 727 (Ser727) and phosphorylation of STAT3 Tyrosine 705 (Tyr705) were both increased by IL-13. It is worth noting that the phosphorylation level of STAT3 Ser727 in the cytoplasm did not increase after LPS stimulation. In addition, the leakage of cytochrome C from mitochondria was increased after LPS stimulation, and IL-13 reduced this leakage (Figures 5C,D). Subsequently, mitochondrial protein expression was further confirmed by Western blot. Phosphorylation of STAT3 Tyr705 was not detected in the protein extracted from mitochondria. However, the phosphorylation of STAT3 Ser727 was increased by IL-13 in the mitochondrion (Figures 5E,F).

### Change of ILC2 in the Myocardial Tissue After Lipopolysaccharide Treatment Was Correlated With the Change of IL-13

**FIGURE 6:**
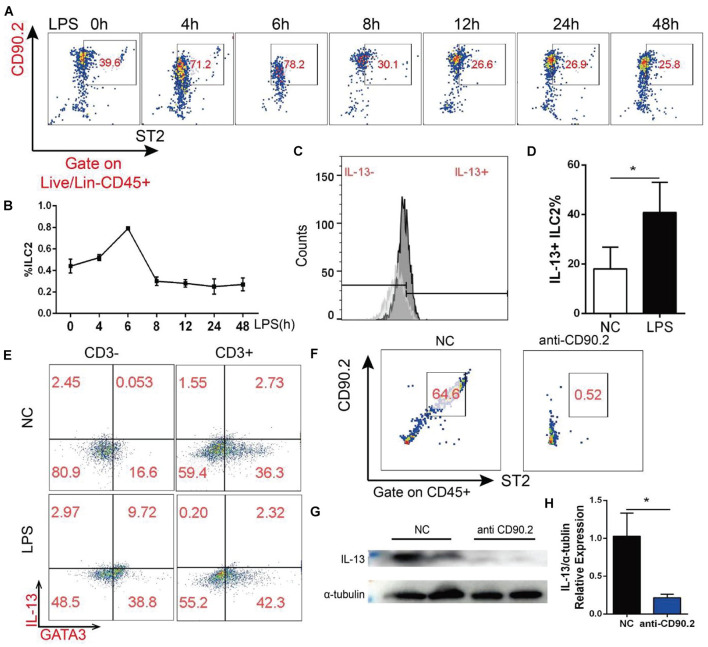
ILC2 change in the myocardial tissue after LPS treatment was correlated with the change of IL-13. **(A)** Change of ILC2 in the myocardial tissue after LPS treatment. **(B)** Quantification of **(A)**. **(C)** Change of IL-13 positive ILC2 in the myocardial tissue of mice treated with LPS. **(D)** Quantification of **(B)**. **(E)** IL-13 secreted by CD3-negative or CD3-positive T cells; **(F)** CD90.2 antibody depletion efficiency of ILC2. **(G)** Expression of IL-13 in the myocardial tissue of mice after CD90.2 antibody treatment. **(H)** Quantification of **(G)**. Data were expressed as the mean ± SD (*n* = 3). *p < 0.05.

As ILC2 is one of the main sources of IL-13, we detected ILC2 change in the heart of mice treated with LPS by flow cytometry. The mouse heart tissue was first processed into single cells and incubated with fluorescence-labeled antibodies, and then ILC2 was further gated as Lineage-, CD45^+^, CD90.2^+^, and ST2^+^ cell (Supplementary Figure 3). As shown in Figures 6A,B, the percentage of ILC2 exhibited a time-dependent change, and this trend was similar to that of IL-13. In addition, LPS increased IL-13 positive ILC2 in the heart (Figures 6C,D). ILC2 is a CD3 negative T cell, and IL-13 in the heart appeared to be mainly derived from CD3 negative cells (Figure 6E). After treating the mice with CD90.2 antibody, ILC2 in the heart was almost completely eliminated (Figure 6F). At the same time, IL-13 of the myocardial tissue was almost cleared (Figures 6G,H). Interestingly, after ILC2 was cleared with CD90.2 antibody, not only IL-13 was reduced but the phosphorylation of STAT3 Ser727 was also inhibited. In contrast, the phosphorylation of STAT3 Tyr705 was enhanced by LPS (Supplementary Figure 4).

## Discussion

During sepsis, activation of type 1 immune response leads to the release of abundant cytokines, which is recognized as the common feature of sepsis, known as “cytokine storm” (Virga et al., 2021). Intriguingly, activation of anti-inflammatory type 2 immune response can antagonize proinflammatory type 1 immunity (Verhoef et al., 2019). A retrospective study observed that pre-existing type 2 immune activation could prevent the progression of sepsis (Krishack et al., 2017). Type 2 immunity is commonly observed in tissues repair and allergic inflammation as represented by the production of type 2 cytokines such as IL-4, IL-5, IL-9, and IL-13 (Flayer et al., 2021). Lai et al. showed that IL-9 derived from ILC2 mediated type 2 immune response and reduced inflammation following sepsis (Lai et al., 2018). In addition, IL-33, IL-13, and IL-5 have also been reported to prevent acute lung injury during sepsis (Linch et al., 2012; Nascimento et al., 2017; Califano et al., 2018). There is evidence that the protective effects of these type 2 cytokines are related to ILC2. The aim of the present study was to explore the effect of IL-13 on the heart during sepsis.

IL-13 has been extensively studied as a classic type 2 cytokine for its effect on immune cells, and the direct effect of IL-13 on target tissue cells has also been explored more recently (Heredia et al., 2013; Wynn, 2015). Some studies reported that IL-13 level was increased after sepsis, while other studies argued that IL-13 was decreased after sepsis (Matsukawa et al., 2000; Akama et al., 2021). The possible reason for this discrepancy is that the specific time points of concern may be different between these studies. A more unified view is that IL-13 plays a protective role during sepsis. In the present study, we detected the expression level of IL-13 in the heart after sepsis over time and found that the expression of IL-13 was not simply a phenomenon of increase or decrease in the early stages of sepsis. Besides, unlike protein expression, the transcription level of IL-13 has always been increased. We found that the serum markers of myocardial injury were noted to return to the normal level 12 h after sepsis, when IL-13 transcription was further enhanced but protein expression was decreased. A possible explanation is that IL-13 is over-consumed. The results of this study showed that IL-13 protected cardiomyocytes against apoptosis during sepsis. IL-13 is known to polarize macrophages toward M2 phenotype, and this effect is beneficial to the survival of cardiomyocytes. To determine whether IL-13 had a direct effect on cardiomyocytes, primary cardiomyocytes were studied *in vitro*. The results showed that IL-13 directly inhibited cardiomyocyte apoptosis caused by LPS.

IL-13 is generally considered to function by activating STAT3 or STAT6 (Yu et al., 2018). However, our RNA-seq showed that Stat6 was not significantly changed in the myocardial tissue. STAT6 signaling has been described as transient in the field of heart research, whereas STAT3 activation is required for cardiomyocytes in response to injury (Fang et al., 2013). In this work, the treatment of rIL-13 did increase the phosphorylation of STAT3 and reduced the pro-apoptotic activity of cleaved-Caspase-3. However, the phosphorylation of STAT3 ser727 did not increase correspondingly in the cytosol. Cytochrome C plays a vital role in the mitochondrial electron transport chain in normal cells (Kalpage et al., 2019). It is reported that Cytochrome C in the cytosol resulted in a positive-feedback mechanism, causing more Cytochrome C release and activation of apoptosis (Li et al., 2000). Thus, the phenomenon that IL-13 suppressed cytochrome C in the cytosol attracted our attention. We hypothesized that the phosphorylated STAT3 Ser727 was transferred into the mitochondrion. The result of our experiment demonstrated that phosphorylated STAT3 Ser727 in mitochondria was increased by IL-13. At the same time, phosphorylated STAT3 Tyr705 was almost undetectable. Previous studies showed that the activation of STAT3 protected cardiomyocytes against apoptosis (Wang et al., 2020). This study supports the protective effect of IL-13 on the myocardium to a certain extent based on the activation of STAT3.

As one of the main sources of IL-13, ILC2 is recognized in tissue repair and macrophage activation (Neill et al., 2010; Guo et al., 2012). However, few studies have focused on the source of IL-13 in the heart. Therefore, multi-label flow cytometry was applied to determine the source of IL-13 in the heart. According to the description of previous studies (Meyer et al., 2020), we applied CD90.2 antibody *in vivo* to eliminate ILC2 and found that IL-13 decreased drastically after eliminating ILC2. Based on these results, the main source of IL-13 in the heart is determined to be ILC2.

The *in vivo* application of CD90.2 antibody not only eliminates ILC2 and reduces IL-13 but directly reduces phosphorylated STAT3 Ser727. Combined with the results of the *in vitro* molecular experiments, it is supported that IL-13 activates the phosphorylation of STAT3 Ser727 to exert anti-apoptotic effects.

In summary, our investigations have demonstrated the importance of IL-13 in alleviating myocardial apoptosis caused by sepsis. IL-13 increases the phosphorylation of STAT3 Ser727 and transfers it into the mitochondria, and reduces the release of cytochrome C. In addition, IL-13 in the heart is derived from ILC2, and the absence of ILC2 will make this protective effect disappear. All these results suggest that IL-13 has therapeutic potential in cardiomyopathy caused by sepsis.

## Data Availability Statement

The original contributions presented in the study are included in the article/Supplementary Material, further inquiries can be directed to the corresponding author/s.

## Ethics Statement

The animal study was reviewed and approved by Ethics Committee of Shanghai Chest Hospital.

## Author Contributions

TH, SL, and XG conducted the experiments. YW, JZ, and XS analyzed the data. MiaZ wrote the manuscript. HJ, QM, and LS designed the manuscript. MinZ and BH designed the methodology and supervised the study. All authors contributed to the article and approved the submitted version.

## Conflict of Interest

The authors declare that the research was conducted in the absence of any commercial or financial relationships that could be construed as a potential conflict of interest.

## Publisher’s Note

All claims expressed in this article are solely those of the authors and do not necessarily represent those of their affiliated organizations, or those of the publisher, the editors and the reviewers. Any product that may be evaluated in this article, or claim that may be made by its manufacturer, is not guaranteed or endorsed by the publisher.

## References

[B1] Akama Y. Park E. J. Satoh-Takayama N. Gaowa A. Ito A. Kawamoto E. (2021). Sepsis induces deregulation of IL-13 production and PD-1 expression in lung group 2 innate lymphoid cells. *Shock* 55 357–370. 10.1097/shk.0000000000001647 32826811

[B2] Califano D. Furuya Y. Roberts S. Avram D. McKenzie A. N. J. Metzger D. W. (2018). IFN-γ increases susceptibility to influenza a infection through suppression of group II innate lymphoid cells. *Mucosal Immunol.* 11 209–219. 10.1038/mi.2017.41 28513592 PMC5693789

[B3] Ehrman R. R. Sullivan A. N. Favot M. J. Sherwin R. L. Reynolds C. A. Abidov A. (2018). Pathophysiology, echocardiographic evaluation, biomarker findings, and prognostic implications of septic cardiomyopathy: a review of the literature. *Crit. Care* 22:112. 10.1186/s13054-018-2043-8 29724231 PMC5934857

[B4] Fang Y. Gupta V. Karra R. Holdway J. E. Kikuchi K. Poss K. D. (2013). Translational profiling of cardiomyocytes identifies an early Jak1/Stat3 injury response required for zebrafish heart regeneration. *Proc. Natl. Acad. Sci. U.S.A.* 110 13416–13421. 10.1073/pnas.1309810110 23901114 PMC3746860

[B5] Flayer C. H. Perner C. Sokol C. L. (2021). A decision tree model for neuroimmune guidance of allergic immunity. *Immunol. Cell Biol.* 10.1111/imcb.12486 [Epub ahead of print]. 34115905 PMC8490305

[B6] Gause W. C. Wynn T. A. Allen J. E. (2013). Type 2 immunity and wound healing: evolutionary refinement of adaptive immunity by helminths. *Nat. Rev. Immunol.* 13 607–614. 10.1038/nri3476 23827958 PMC3789590

[B7] Gieseck R. L. III Wilson M. S. Wynn T. A. (2018). Type 2 immunity in tissue repair and fibrosis. *Nat. Rev. Immunol.* 18 62–76. 10.1038/nri.2017.90 28853443

[B8] Guo L. Junttila I. S. Paul W. E. (2012). Cytokine-induced cytokine production by conventional and innate lymphoid cells. *Trends Immunol.* 33 598–606. 10.1016/j.it.2012.07.006 22959641 PMC4799496

[B9] Heredia J. E. Mukundan L. Chen F. M. Mueller A. A. Deo R. C. Locksley R. M. (2013). Type 2 innate signals stimulate fibro/adipogenic progenitors to facilitate muscle regeneration. *Cell* 153 376–388. 10.1016/j.cell.2013.02.053 23582327 PMC3663598

[B10] Hofmann U. Knorr S. Vogel B. Weirather J. Frey A. Ertl G. (2014). Interleukin-13 deficiency aggravates healing and remodeling in male mice after experimental myocardial infarction. *Circ. Heart Fail.* 7 822–830. 10.1161/circheartfailure.113.001020 24970469

[B11] Hong T. Wei Y. Xue X. Li Y. Dong H. Guo X. (2020). A novel anti-coagulative nanocomplex in delivering miRNA-1 inhibitor against microvascular obstruction of myocardial infarction. *Adv. Healthc. Mater.* 9:e1901783. 10.1002/adhm.201901783 32338452

[B12] Joseph L. C. Kokkinaki D. Valenti M. C. Kim G. J. Barca E. Tomar D. (2017). Inhibition of NADPH oxidase 2 (NOX2) prevents sepsis-induced cardiomyopathy by improving calcium handling and mitochondrial function. *JCI Insight* 2:e94248. 10.1172/jci.insight.94248 28878116 PMC5621873

[B13] Kalpage H. A. Bazylianska V. Recanati M. A. Fite A. Liu J. Wan J. (2019). Tissue-specific regulation of cytochrome c by post-translational modifications: respiration, the mitochondrial membrane potential, ROS, and apoptosis. *FASEB J.* 33 1540–1553. 10.1096/fj.201801417R 30222078 PMC6338631

[B14] Kamisoglu K. Haimovich B. Calvano S. E. Coyle S. M. Corbett S. A. Langley R. J. (2015). Human metabolic response to systemic inflammation: assessment of the concordance between experimental endotoxemia and clinical cases of sepsis/SIRS. *Crit. Care* 19:71. 10.1186/s13054-015-0783-2 25887472 PMC4383069

[B15] Krishack P. A. Wang K. Rzhetsky A. Solway J. Sperling A. I. Verhoef P. A. (2017). Preexisting type 2 immune activation protects against the development of sepsis. *Am. J. Respir. Cell Mol. Biol.* 57 628–630. 10.1165/rcmb.2017-0277LE 29090959 PMC5705910

[B16] Lai D. Tang J. Chen L. Fan E. Scott M. Li Y. (2018). Group 2 innate lymphoid cells protect lung endothelial cells from pyroptosis in sepsis. *Cell Death Dis.* 9:369. 10.1038/s41419-018-0412-5 29511181 PMC5840374

[B17] Li K. Li Y. Shelton J. M. Richardson J. A. Spencer E. Chen Z. J. (2000). Cytochrome c deficiency causes embryonic lethality and attenuates stress-induced apoptosis. *Cell* 101 389–399. 10.1016/s0092-8674(00)80849-1 10830166

[B18] Linch S. N. Danielson E. T. Kelly A. M. Tamakawa R. A. Lee J. J. Gold J. A. (2012). Interleukin 5 is protective during sepsis in an eosinophil-independent manner. *Am. J. Respir. Crit. Care Med.* 186 246–254. 10.1164/rccm.201201-0134OC 22652030 PMC3423456

[B19] Matsukawa A. Hogaboam C. M. Lukacs N. W. Lincoln P. M. Evanoff H. L. Strieter R. M. (2000). Expression and contribution of endogenous IL-13 in an experimental model of sepsis. *J. Immunol.* 164 2738–2744. 10.4049/jimmunol.164.5.2738 10679115

[B20] Meyer A. R. Engevik A. C. Madorsky T. Belmont E. Stier M. T. Norlander A. E. (2020). Group 2 innate lymphoid cells coordinate damage response in the stomach. *Gastroenterology* 159 2077–2091.e8. 10.1053/j.gastro.2020.08.051 32891625 PMC7726005

[B21] Nascimento D. C. Melo P. H. Piñeros A. R. Ferreira R. G. Colón D. F. Donate P. B. (2017). IL-33 contributes to sepsis-induced long-term immunosuppression by expanding the regulatory T cell population. *Nat. Commun.* 8:14919. 10.1038/ncomms14919 28374774 PMC5382289

[B22] Neill D. R. Wong S. H. Bellosi A. Flynn R. J. Daly M. Langford T. K. (2010). Nuocytes represent a new innate effector leukocyte that mediates type-2 immunity. *Nature* 464 1367–1370. 10.1038/nature08900 20200518 PMC2862165

[B23] O’Meara C. C. Wamstad J. A. Gladstone R. A. Fomovsky G. M. Butty V. L. Shrikumar A. (2015). Transcriptional reversion of cardiac myocyte fate during mammalian cardiac regeneration. *Circ. Res.* 116 804–815. 10.1161/circresaha.116.304269 25477501 PMC4344930

[B24] Verhoef P. A. Bhavani S. V. Carey K. A. Churpek M. M. (2019). Allergic immune diseases and the risk of mortality among patients hospitalized for acute infection. *Crit. Care Med.* 47 1735–1742. 10.1097/ccm.0000000000004020 31599813 PMC6861689

[B25] Virga F. Cappellesso F. Stijlemans B. Henze A. T. Trotta R. Van Audenaerde J. (2021). Macrophage miR-210 induction and metabolic reprogramming in response to pathogen interaction boost life-threatening inflammation. *Sci. Adv.* 7:eabf0466. 10.1126/sciadv.abf0466 33962944 PMC7616432

[B26] Wang Q. Zhu Q. Ye Q. Wang J. Dong Q. Chen Y. (2020). STAT3 suppresses cardiomyocytes apoptosis in CVB3-induced myocarditis via survivin. *Front. Pharmacol.* 11:613883. 10.3389/fphar.2020.613883 33658937 PMC7919905

[B27] Wodsedalek D. J. Paddock S. J. Wan T. C. Auchampach J. A. Kenarsary A. Tsaih S. W. (2019). IL-13 promotes in vivo neonatal cardiomyocyte cell cycle activity and heart regeneration. *Am. J. Physiol. Heart Circ. Physiol.* 316 H24–H34. 10.1152/ajpheart.00521.2018 30339498

[B28] Wynn T. A. (2015). Type 2 cytokines: mechanisms and therapeutic strategies. *Nat. Rev. Immunol.* 15 271–282. 10.1038/nri3831 25882242

[B29] Yu Q. N. Guo Y. B. Li X. Li C. L. Tan W. P. Fan X. L. (2018). ILC2 frequency and activity are inhibited by glucocorticoid treatment via STAT pathway in patients with asthma. *Allergy* 73 1860–1870. 10.1111/all.13438 29542140 PMC6175310

[B30] Zechendorf E. O’Riordan C. E. Stiehler L. Wischmeyer N. Chiazza F. Collotta D. (2020). Ribonuclease 1 attenuates septic cardiomyopathy and cardiac apoptosis in a murine model of polymicrobial sepsis. *JCI Insight* 5:e131571. 10.1172/jci.insight.131571 32213712 PMC7205433

[B31] Zlatanova I. Pinto C. Bonnin P. Mathieu J. R. R. Bakker W. Vilar J. (2019). Iron regulator hepcidin impairs macrophage-dependent cardiac repair after injury. *Circulation* 139 1530–1547. 10.1161/circulationaha.118.034545 30586758

