# Supplementary material for: IL-13 Derived Type 2 Innate Lymphocytes Ameliorates Cardiomyocyte Apoptosis Through STAT3 Signaling Pathway
Source: Front Cell Dev Biol. 2021 Sep 20;9:742662. doi: 10.3389/fcell.2021.742662 (PMC8488199; doi:10.3389/fcell.2021.742662)
Supplement: Supplementary file 1 [file Data_Sheet_1.doc]

IL-13 Derived Type 2 Innate Lymphocytes Ameliorates Cardiomyocyte Apoptosis through STAT3 Signaling Pathway

Supplementary Material

**Supplemental Table 1. List of fluorescent labeled antibodies**

| **Name** | **Fluorescence** | **Host Species** |
| --- | --- | --- |
| Anti-mouse CD11c | BV605 | Rat |
| Anti-mouse CD127 | BV421 | Rat |
| Anti-mouse CD3 | BV785 | Rat |
| Anti-mouse CD3 | APC/Cy7 | Rat |
| Anti-mouse CD45 | PerCP/Cy5.5 | Rat |
| Anti-mouse CD45 | FITC | Rat |
| Anti-mouse CD90.2 | AF700 | Rat |
| Anti-mouse GATA3 | APC | Rat |
| Anti-mouse GATA3 | AF488 | Rat |
| Anti-mouse Gr-1 | BV605 | Rat |
| Anti-mouse IL-13 | PE | Rat |
| Anti-mouse IL-13 | PE/Cy7 | Rat |
| Anti-mouse Lineage | FITC | Armenian Hamster |
| Anti-mouse ST2 | APC | Rat |
| Anti-mouse ST2 | AF750 | Rat |
| Anti-mouse/Human B220 | BV605 | Rat |
| Zombie Green™ Fixable Viability Kit | / | mouse |
| Zombie UV™ Fixable Viability Kit | / | mouse |


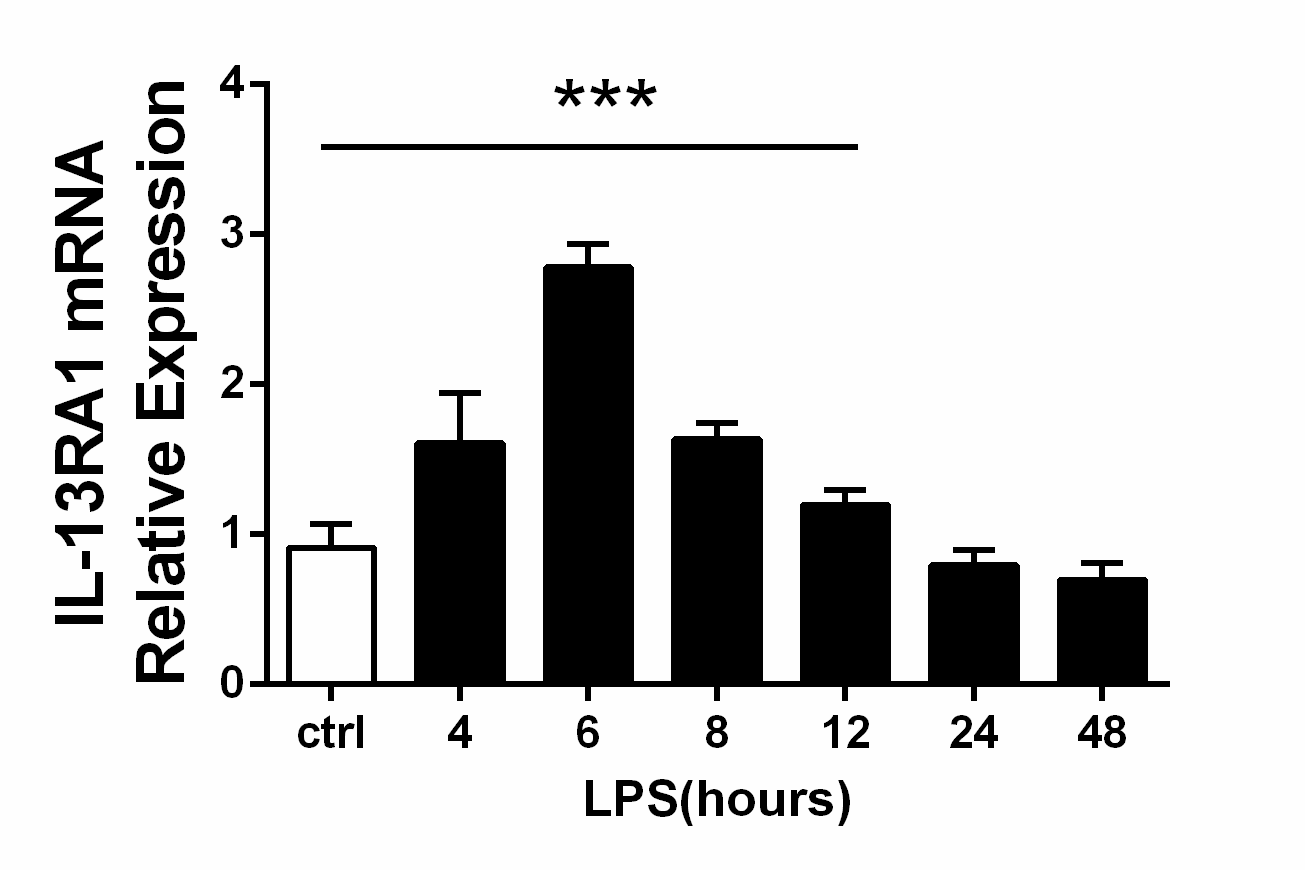


**Figure S1** mRNA expression of IL-13Rα1 within 48 hours after LPS intraperitoneal injection in the myocardial tissue measured by qPCR. Data were expressed as the mean±SD (n=6). *p<0.05, **p<0.01, ***p<0.001.


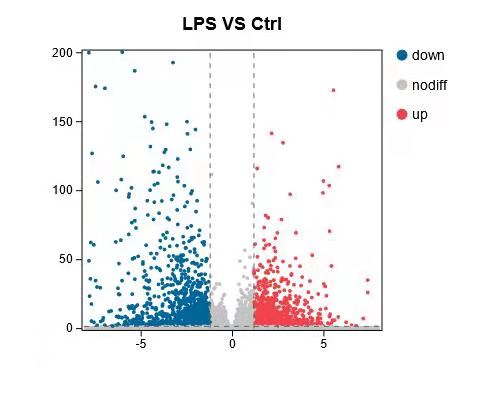
**
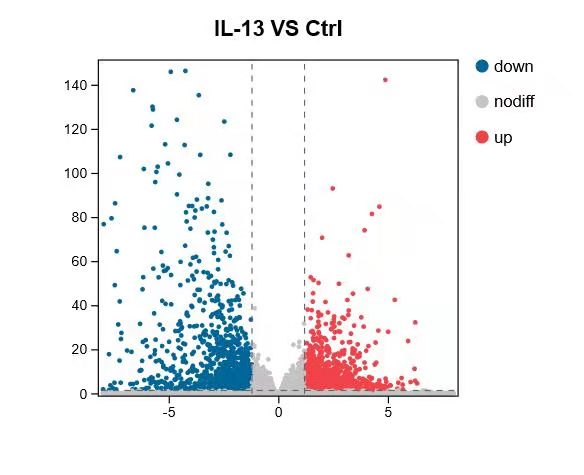
**

**Figure S2** (**A**) Volcano picture of differential expressed genes (DEGs) of LPS group versus Control group . P<0.05, |Log_2_FC|>1.2; (**B**) Volcano picture of differential expressed genes (DEGs) of IL-13 group versus LPS group . P<0.05, |Log_2_FC|>1.2.(n=6)


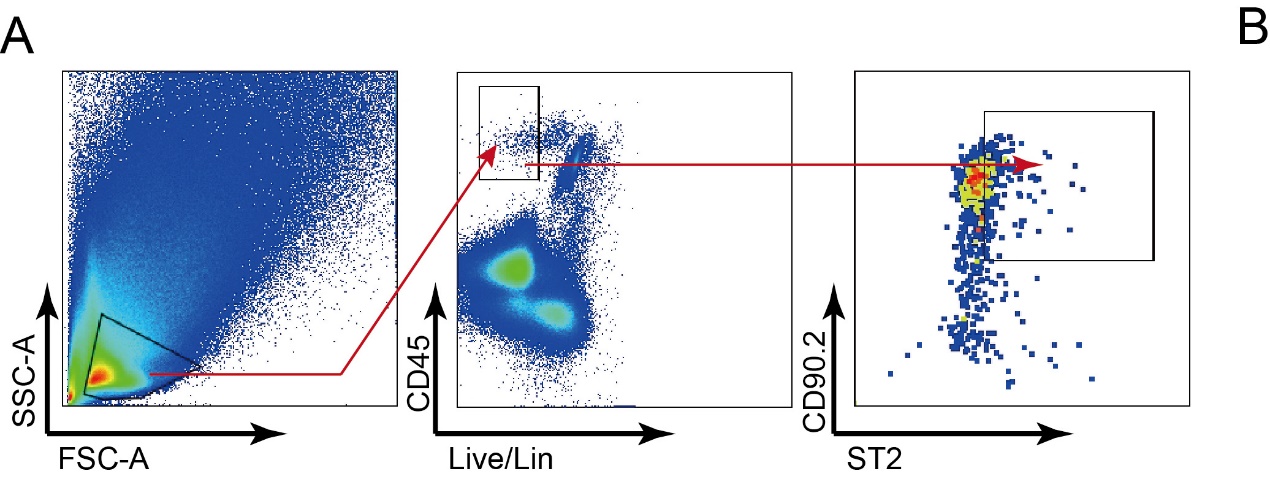


**Figure S3** Gate strategy for ILC2 in heart tissue.


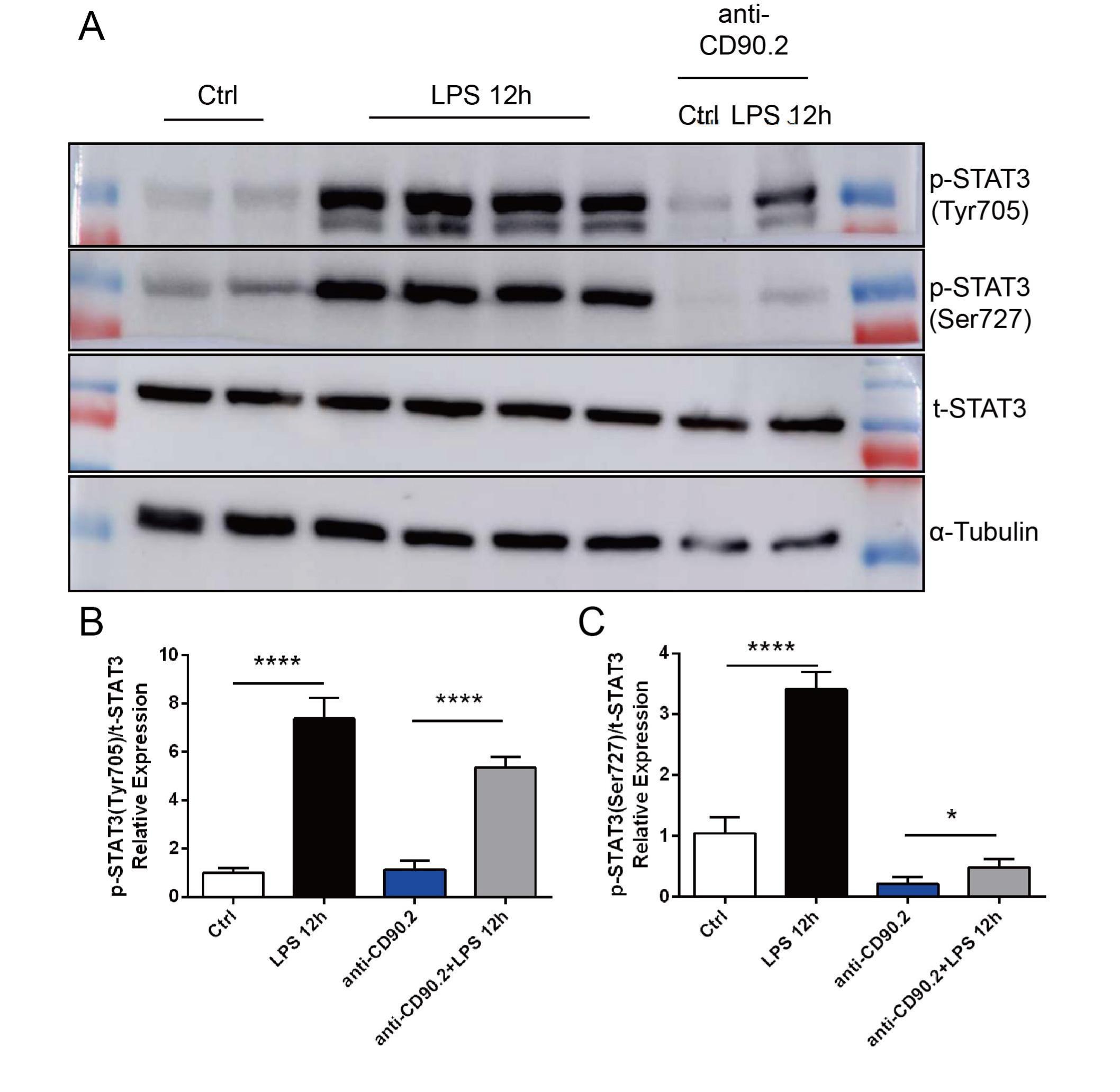


**Figure S4** (**A**) phosphorylation of STAT3 in heart tissue detected by Western Blot; (**B,C**) Quantification of A. Data are expressed as the mean±SD (n=3). *p<0.05, **p<0.01, ***p<0.001, ****p<0.0001.

**
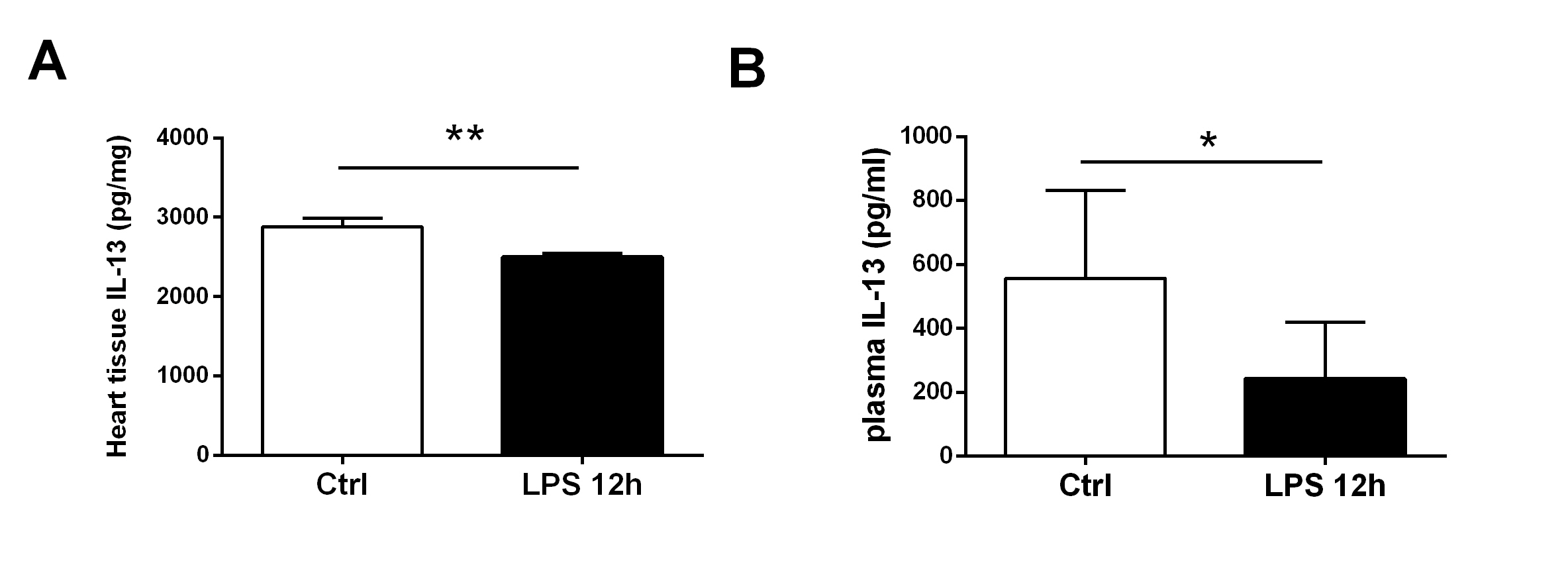
Figure S5 (A)** Heart tissue IL-13 measured by ELISA at 12 hours after LPS intraperitoneal injection; **(B)** Separated plasma IL-13 measured by ELISA at 12 hours after LPS intraperitoneal injection. Data were expressed as the mean±SD (n=3). *p<0.05, **p<0.01.

**Supplemental Methods**

**Echocardiographic assessment of cardiac function**

Transthoracic echocardiography with an ultrasound instrument (Vivid 7 GE Medical) equipped with a 10-MHz phased-array transducer was used to measure the Left ventricle function. Left ventricle ejection fraction (LVEF) and fractional shortening (FS) were calculated from M-mode recording.

**TUNEL staining**

The heart was fixed with 4% paraformaldehyde and embedded in paraffin, and then sectioned for further detection. After the slices were slightly dried, the membrane breaking working solution was added dropwise, incubated at room temperature for 20min, and then washed 3 times for 5min each time. The tissues were incubated with TUNEL reaction buffer at room temperature for 10 minutes, then TDT enzyme, dUTP and buffer were mixed in the ratio of 1:5:50. After adding them to the tissues, the reaction continued at room temperature for another 2 hours. The nuclei was stained with DAPI and then sealed.

**Immunofluorescent Staining**

## Following the dehydration, embedding, and deparaffinization process, the heart tissues were evenly sliced into sections and subjected to immunofluorescent staining. In simple terms, slides were subjected to Permeabilization and then take a blocking step for 15 min with BSA. After washing with DPBS, slides were incubated for 30 min with a [PE anti-mouse IL-13 Antibody](https://www.biolegend.com/en-us/search-results/pe-anti-mouse-il-13-antibody-19159)(Biolegend,USA) and eventually rinsed with DPBS and air-dried. All samples were additionally stained with DAPI to identify cells by nuclei visualization.

**Immunohistochemical Staining**

After deparaffinization, the sections undergo an antigen retrieval process, and treated with 3% hydrogen peroxide and blocked with serum. After the primary antibody was incubated overnight at 4°C, the secondary antibody was incubated at room temperature. The color of the labeled antibody is determined by a chemical reaction to determine the color of the antigen in the tissue cell. Finally, hematoxylin is used to make the nucleus visible.
